# Early response evaluation using primary tumor and nodal imaging features to predict progression-free survival of locally advanced non-small cell lung cancer

**DOI:** 10.7150/thno.50565

**Published:** 2020-09-23

**Authors:** Nasha Zhang, Rachel Liang, Michael F. Gensheimer, Meiying Guo, Hui Zhu, Jinming Yu, Maximilian Diehn, Bill W Loo, Ruijiang Li, Jia Wu

**Affiliations:** 1 Department of Radiation Oncology, Stanford University School of Medicine, 1070 Arastradero Rd, Palo Alto, CA 94304; 2 Department of Radiation Oncology, Shandong Cancer Hospital and Institute, Shandong First Medical University and Shandong Academy of Medical Sciences, Jinan, China; 3 Imaging Physics, Thoracic/Head & Neck Medical Oncology (joint appointment), MD Anderson Cancer Center, 1400 Pressler St., Unit 1472, Houston, Texas 77030

**Keywords:** locally advanced NSCLC, pre and mid-treatment PET, radiomics, imaging model, PFS

## Abstract

Prognostic biomarkers that can reliably predict early disease progression of non-small cell lung cancer (NSCLC) are needed for identifying those patients at high risk for progression, who may benefit from more intensive treatment. In this work, we aimed to identify an imaging signature for predicting progression-free survival (PFS) of locally advanced NSCLC.

**Methods**: This retrospective study included 82 patients with stage III NSCLC treated with definitive chemoradiotherapy for whom both baseline and mid-treatment PET/CT scans were performed. They were randomly placed into two groups: training cohort (n=41) and testing cohort (n=41). All primary tumors and involved lymph nodes were delineated. Forty-five quantitative imaging features were extracted to characterize the tumors and involved nodes at baseline and mid-treatment as well as differences between two scans performed at these two points. An imaging signature was developed to predict PFS by fitting an L1-regularized Cox regression model.

**Results**: The final imaging signature consisted of three imaging features: the baseline tumor volume, the baseline maximum distance between involved nodes, and the change in maximum distance between the primary tumor and involved nodes measured at two time points. According to multivariate analysis, the imaging model was an independent prognostic factor for PFS in both the training (hazard ratio [HR], 1.14, 95% confidence interval [CI], 1.04-1.24; *P* = 0.003), and testing (HR, 1.21, 95% CI, 1.10-1.33; *P* = 0.048) cohorts. The imaging signature stratified patients into low- and high-risk groups, with 2-year PFS rates of 61.9% and 33.2%, respectively (*P* = 0.004 [log-rank test]; HR, 4.13, 95% CI, 1.42-11.70) in the training cohort, as well as 43.8% and 22.6%, respectively (*P* = 0.006 [log-rank test]; HR, 3.45, 95% CI, 1.35-8.83) in the testing cohort. In both cohorts, the imaging signature significantly outperformed conventional imaging metrics, including tumor volume and SUV_max_ value (C-indices: 0.77-0.79 for imaging signature, and 0.53-0.73 for conventional metrics).

**Conclusions**: Evaluation of early treatment response by combining primary tumor and nodal imaging characteristics may improve the prediction of PFS of locally advanced NSCLC patients.

## Introduction

Lung cancer ranks first in cancer-related deaths throughout the world 1. Non-small cell lung cancer (NSCLC) accounts for about 85% of all lung cancer cases and is a rapidly proliferating tumor. Thirty percent of NSCLCs are diagnosed at a locally advanced TNM stage (stage III) 2, for which concurrent chemoradiotherapy (CCRT) is the clinical standard treatment regimen according to National Comprehensive Cancer Network guidelines 3. Nevertheless, the median overall survival remains poor for locally advanced NSCLC (10-14 months) 4. In these patients, tumor heterogeneity is a crucial factor for poor prognosis, with intra-tumoral heterogeneity and tumor evolution resulting in therapy resistance and disease progression 5. Even in patients diagnosed at identical clinical stages, the distinct biological components and evolution trajectories of individual tumors lead to significant discrepancies in their response to standard treatment regimens 5, 6. Therefore, biomarkers that can effectively predict treatment failure at individual patient level are of great value for precisely treating them. Results of a phase 3 randomized clinical trial (RTOG 0617) underscored the importance of prognostic biomarkers. In that study, patients with locally advanced NSCLC who received high-dose radiotherapy (RT) did not have a survival benefit when compared with those receiving standard-dose. Contrary to the original hypothesis of the trial, escalating RT dose or adding cetuximab for all enrolled stage III NSCLCs was harmful, whereas adverse events of grade 3 or higher adverse events occurred in 79% of cases in treatment intensified group 7. In such situations, reliable biomarkers are urgently needed to identify NSCLC patients at high risk for disease progression and who may benefit from a RT dose boost or other intensive treatments.

In recent years, ^18^F-fluorodeoxyglucose (^18^F-FDG) positron emission tomography (PET) has been increasingly applied to clinical management of NSCLC, including diagnosis, staging, target volume delineation before RT, treatment response assessment, and progression risk stratification for patients 8-10. In preliminary studies, some univocal imaging features of PET, such as the maximum standardized uptake value (SUV_max_) and metabolic tumor volume (MTV), demonstrated prognostic values for NSCLC 11-15. However, these results were not universally consistent. For example, according to the ACRIN 6668/RTOG 0235 trial, pretreatment MTV as assessed by ^18^F-FDG PET/CT is a prognostic factor in patients with stage III NSCLC after definitive chemoradiation 11. However, Guberina et al. failed to validate this result in the German phase 3 trial ESPATUE, and they reported no differences in the survival curves for patients with high and low MTVs 12. Additionally, treatment individualization according to MTV was not supported by this study 12. However, Huang et al. studied 53 patients with stage IIIA-IIIB NSCLC who underwent radical chemoradiotherapy and reported that decreased MTV during treatment was associated with improved overall survival (OS) 14. Furthermore, in a study by Kong et al., a greater reduction in mid-treatment ^18^F-FDG PET volume predicted worse survival of locally advanced NSCLC 15. Possible reasons accounting for these conflicting results are that simple volumetric features may not represent the landscape of complicated, heterogeneous NSCLC tumors. In addition, up to date, most of radiomics studies have focused on the analysis of primary tumors and neglected involved lymph nodes, which contain critical information for staging and treatment planning and are closely linked with prognosis for stage III NSCLC 16.

Computational radiomics analysis has extracted diverse information from images acquired in routine clinical practice and played increasingly essential roles in the response prediction and survival prognostication for several types of cancer, including NSCLC 17-20. Therefore, in the present work, we sought to determine the prognostic ability of combined pre-RT and mid-RT PET imaging features in stage III NSCLC patients in the most extensive such series reported to date. We hypothesized that a radiomic signature integrating quantitative imaging characteristics of the tumors and lymph nodes extracted from ^18^F-FDG PET and CT scans to depict their longitudinal variations can provide a complete, dynamic evaluation of the disease burden and thus better predict clinical outcomes at the individual level than conventional imaging metrics.

## Methods

### Study design

**Figure 1:**
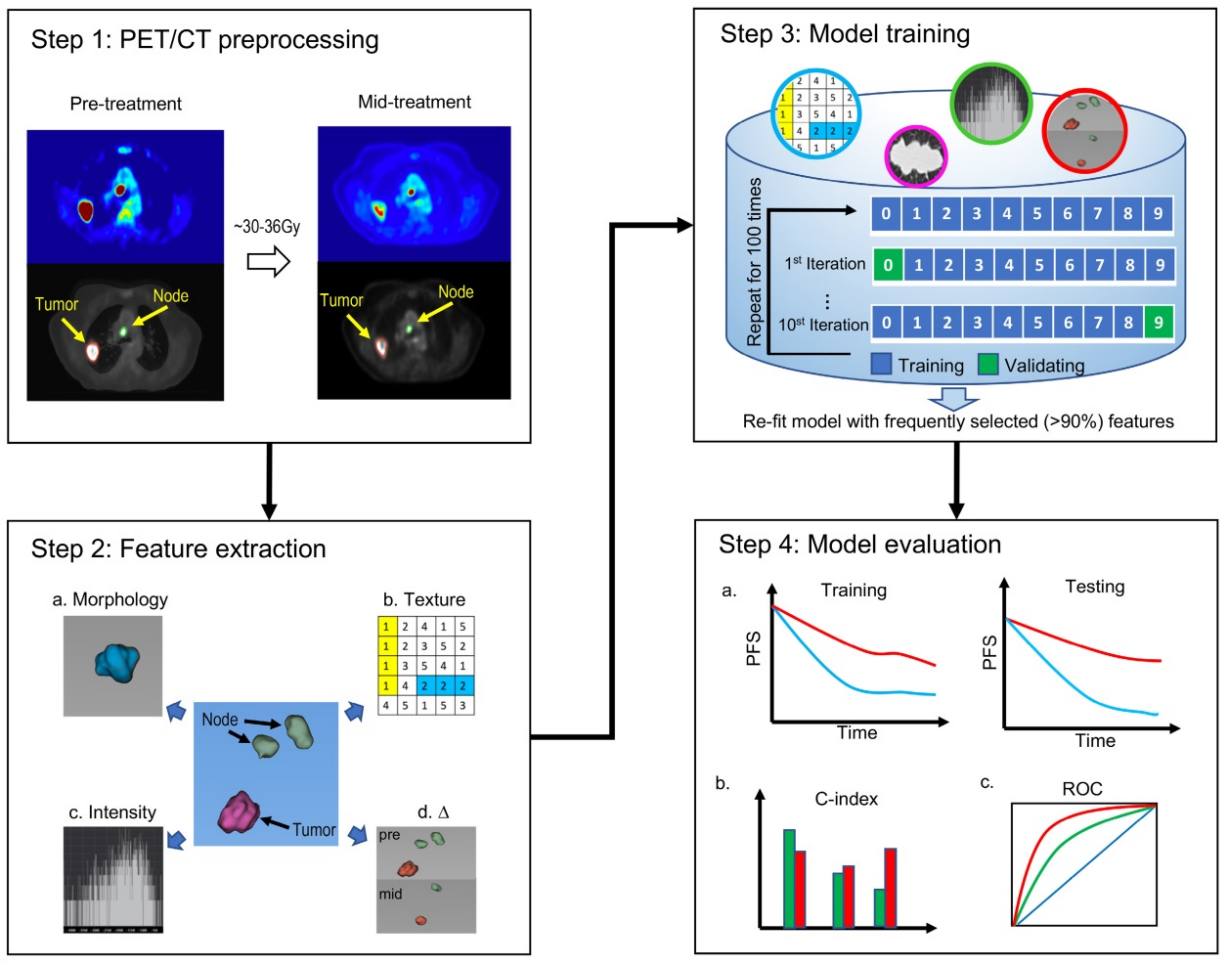
** The overall study design.** The study was conducted in four steps. Step 1: tumors and lymph nodes were segmented and delineated on both baseline and mid-RT fused PET/CT scans. Step 2: quantitative image features were extracted from 3D ROIs. Step 3: we developed an imaging signature to predict progression-free survival by fitting an L1-regularized Cox regression model. Step 4: the model performance was assessed in the training cohort and validated in the testing cohort. The C-index and receiver operating characteristic curve were applied to evaluate the performance of the imaging model. ROI = region of interest.

As Figure 1 shows, this retrospective study was conducted in four steps. First, non-small cell lung tumors and involved nodes were segmented on both baseline and mid-treatment fused ^18^F-FDG PET/CT scans. Second, quantitative image features were extracted from PET/CT scans. Third, an imaging signature was developed to predict progression-free survival (PFS) by fitting an L1-regularized Cox regression model based on a training cohort of the study patients. Fourth, the model performance was assessed in the training cohort and validated in a testing cohort. The C-index and receiver operating characteristic curve (ROC) were applied to evaluate the performance of the imaging model.

### Study population

With Institutional Review Board approval, we retrospectively collected data on patients with stage III NSCLC treated consecutively at Stanford University Medical Center from October 2005 to July 2017. The inclusion criteria were 1) biopsy-confirmed NSCLC, 2) 6-7 weeks of radiation therapy (RT) with concurrent platinum-based doublet chemotherapy, and 3) a baseline ^18^F-FDG PET/CT scan before treatment and a second ^18^F-FDG PET/CT examination half-way through RT. Exclusion criteria included 1) previous induction chemotherapy or surgery, 2) no primary tumor (T0), and 3) incomplete or poor-quality ^18^F-FDG PET/CT. After balancing various clinical and pathological risk factors, patients were equally divided into the training cohort (n=41) and testing cohort (n=41). To mitigate random effects and ensure balanced splitting, patients were stratified by matching them according to age, sex, TNM stage, and Karnofsky performance score (KPS).

### ^18^F-FDG PET/CT image acquisition and segmentation

The PET/CT scans were performed using either a Siemens Biograph mCT scanner (Siemens, Erlangen, Germany) or a GE Discovery scanner (GE Medical Systems, Milwaukee, WI, USA). CT scans were acquired at a tube potential of 120 kV and current of 250 mA. CT images had a slice thickness of 1.25 mm, and in-plane spatial resolution of 1.02×1.02 mm^2^. Patients fasted for at least 8 hours before the examinations to ensure their blood glucose levels were below 180 mg/dL. Afterward, each patient received an intravenous injection of 10-18 mCi of ^18^F-FDG and underwent PET/CT 45-60 min later. The PET images were then reconstructed with time-of-flight and point-spread function modeling or an ordered set expectation maximization algorithm expectation method, with the CT data for attenuation correction. The original PET image spatial resolution was 2.34 × 2.34 × 3.27 mm^3^. The detailed imaging protocols were reported in previous studies 21, 22.

PET/CT images archived in the PACS were exported in DICOM format. The mediastinal staging was evaluated by PET/CT imaging and image-guided biopsy according to the specific location of lymph nodes. The primary tumors and involved lymph nodes were separately delineated on pre-RT and mid-RT PET/CT scans by an attending radiation oncologist specializing in lung cancer. The contour structure data were extracted for further analyses using validated in-house software in MATLAB (MathWorks, Natick, MA, USA) 23.

### Extraction of quantitative image features of primary tumors and involved lymph nodes

High-throughput parameter-based extraction of regions of interest (ROI) on pre-RT and mid-RT PET/CT scans and assessment of the differences between the two scans were performed using MATLAB. All imaging features were calculated based on 3D ROIs. To reduce variability in the PET SUV map, the PET SUV was normalized according to the average background activity in a circular region of interest in the aortic arch. All images were then resampled at an isotropic spatial resolution of 1.0 mm^3^ using the imresize3 function in Matlab (version R2017b).

As shown in Table 1, 45 quantitative imaging features were extracted, including morphology, boundary sharpness, intensity, and gray-level co-occurrence matrix (GLCM) texture features measuring intra-tumoral heterogeneity of primary tumors, as well as volume, border sharpness, and intensity of lymph nodes. A similar set of features has been used in the assessment of breast and head and neck cancer 23, 24. For lymph nodes, three additional features were used to characterize the nodal burden and locoregional invasion of disease: 1) the total number of involved lymph nodes (N_morph.num_); 2) nodal spread, which is the maximum distance among the lymph nodes (N_morph.spread1_); and 3) node-tumor spread, which is the longest distance from the tumor border to the edge of the farthest lymph node (N_morph.spread2_). The changes in tumor volume, lymph node volume, node number, nodal spread, and node-tumor spread during treatment were also calculated as important Δ radiomics features, which reflect the early therapeutic response as determined between the pre-RT and mid-RT PET/CT scans.

### Construction of an imaging signature for predicting NSCLC progression

Based on the 45 quantitative imaging features, a multivariate Cox proportional hazard regression model was fitted to predict PFS in the training cohort. To avoid over-fitting, the Least Absolute Shrinkage and Selection Operator (LASSO) algorithm was used to select features. During this process, the 10-fold cross-validation scheme was performed 100 times to minimize selection bias. The importance of these features was determined during the model construction as to be their selection probability. Image features with a selected frequency greater than 90% were used to re-fit the final imaging model.

### Evaluation of the imaging model

The value of imaging signature in predicting PFS was validated in the hold out testing cohort. The ability to predict 2-year PFS was assessed using survival receiver operating characteristic (ROC) analysis and area under the curve (AUC). In a previous study, the 5-year overall survival rate in stage IIIA NSCLC patients was 19%, whereas that in stage IIIB patients was 7% 4. The performance of the proposed imaging signature was compared to different TNM stage, i.e. stage IIIA versus stage IIIB. Next, the prognostic performance of the proposed imaging signature was compared to conventional PET/CT metrics, including pre-RT tumor volume, mid-RT tumor volume, Δtumor volume, pre-RT SUV_max_, mid-RT SUV_max_, and ΔSUV_max_. The survival data were censored at 2 year as a clinically meaningful endpoint. Moreover, we have carried ablation study to include the proposed imaging features solely from pre-RT PET/CT (n=20) or mid-RT PET/CT (n=20), and repeated the whole process of model construction. The ablated models were compared to the model constructed with all 45 radiomics features.

### Statistics

The Cox proportional hazards model was used to calculate C-index, hazard ratio (HR), and 95% confidence interval (CI). Kaplan-Meier analysis and log-rank test were used for evaluating patient stratification into different risk groups regarding disease progression, where their survival differences were measured by HR and 95% CI. Receiver operating characteristic analysis was applied to evaluate the prognostic accuracy of the predictive models. In these analyses, *P* values less than 0.05 were considered significant, and all statistical tests were two-sided. Statistical analysis was performed in the R (R Foundation, Vienna, Austria).

## Results

### Patients characteristics

**Figure 2:**
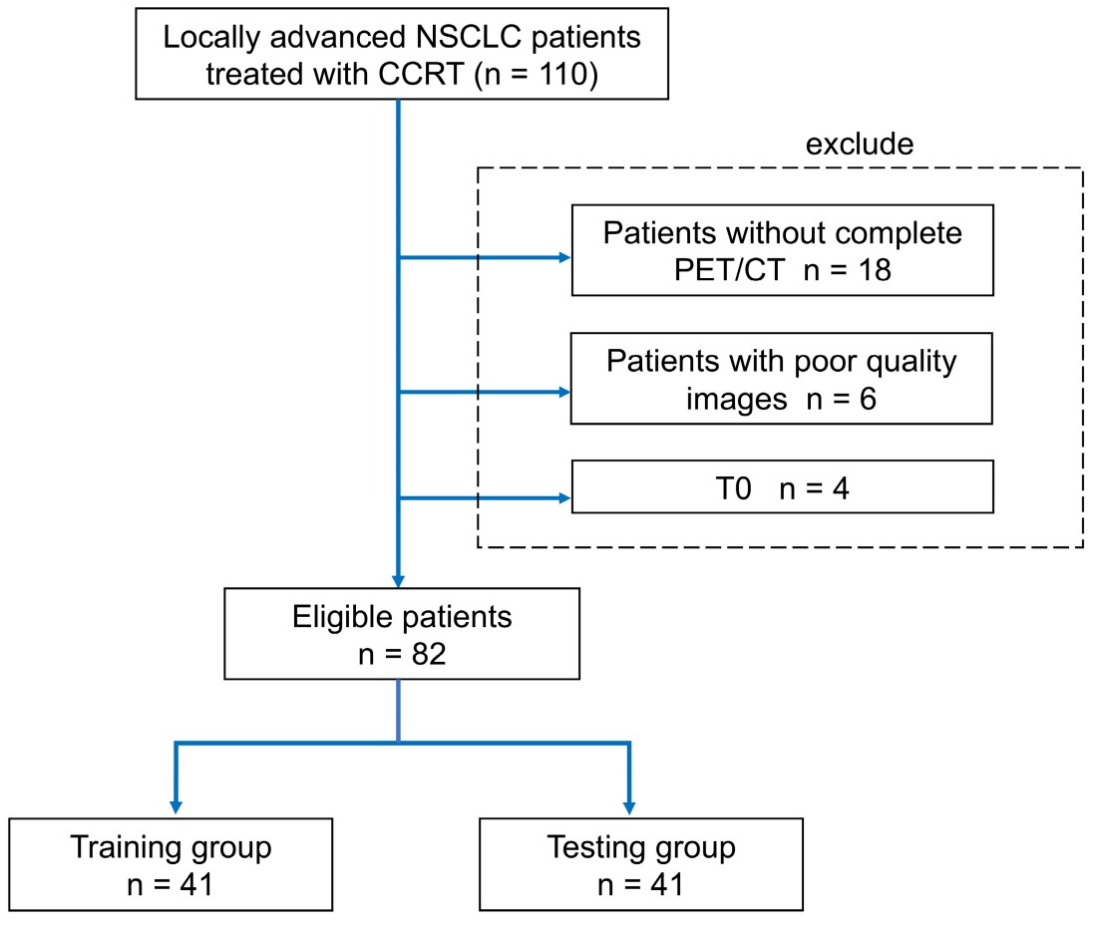
** Protocol enrollment and analysis diagram for the study.** CCRT=concurrent chemoradiotherapy.

Eighty-two patients with stage III NSCLC were enrolled in this study (Figure 2). Patients received an initial radiation doses ranging from 60.0 Gy to 80.4 Gy (median, 66.0 Gy). PET/CT scans were performed half-way through RT for re-planning purpose. By the time of mid-RT PET/CT, the radiation doses ranged from 22.0 Gy to 48.4 Gy. The patients' clinical characteristics are shown in Table 2. In the training cohort, the median age of patients was 68.2 years (range, 41.5-89.2 years). Twenty of these patients (48.8%) had stage IIIA disease, and 21 patients (51.2%) had stage IIIB disease. Forty-four percent of the patients had adenocarcinoma, and 39% of them had squamous cell carcinoma. In the testing group, the median age was 67.9 years (range, 47.2-89.7 years). Eighteen of these patients (43.9%) had stage IIIA disease, and 23 patients (56.1%) had stage IIIB disease. Also, 44% of these patients had adenocarcinoma, and 24.4% had squamous cell carcinoma. At the end of the follow-up period, the disease progression rates were 48.8% in the training cohort and 51.2% in the testing cohort. We did not observe significant differences in the clinical factors between the stratified training and testing cohorts.

### Details on the construction of imaging signature

**Figure 3:**
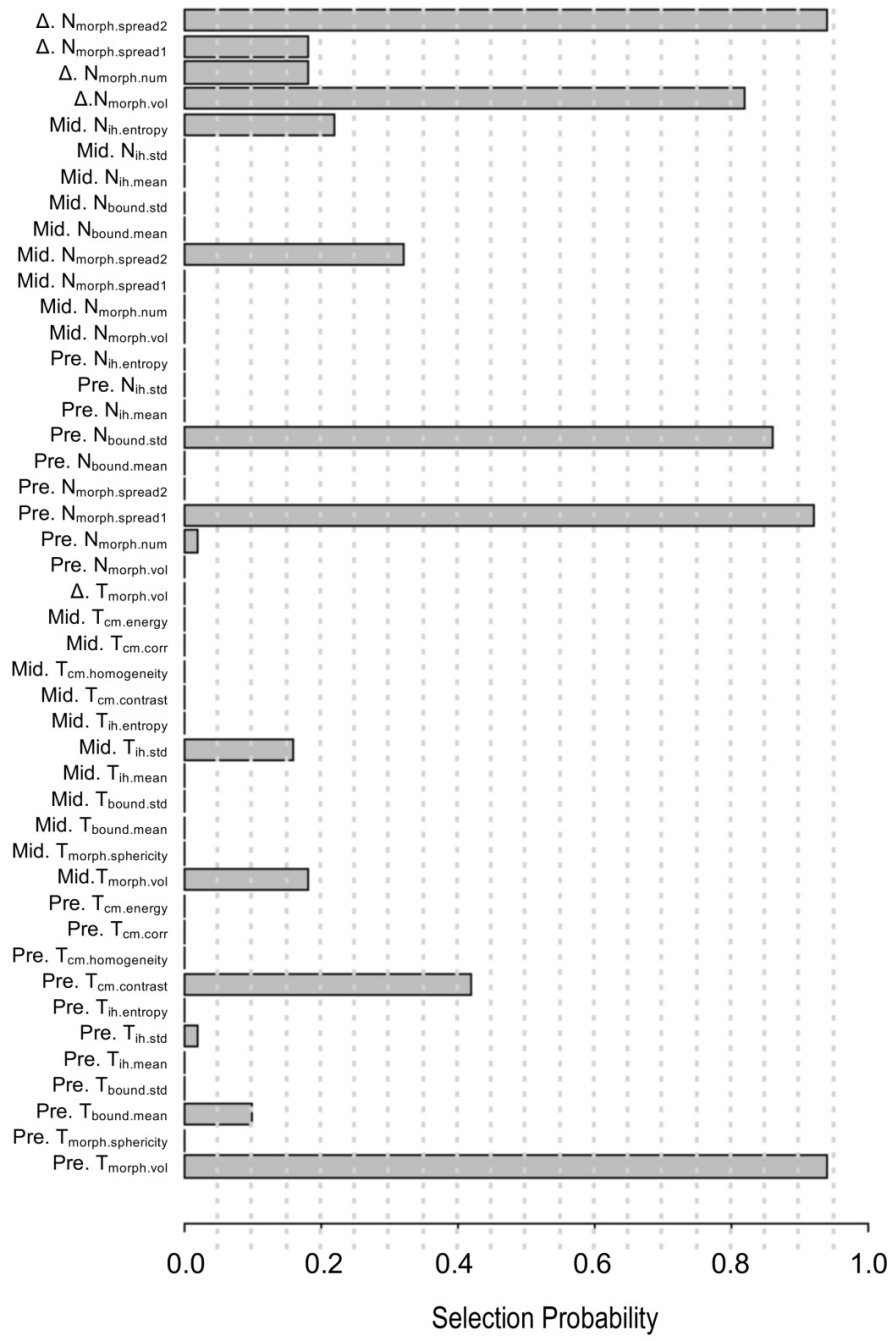
** The 45 quantitative imaging features selected for predicting PFS.** The selection probability represents the importance of individual features.

A pair-wise correlation heat map of the proposed 45 quantitative imaging features showed that these features were largely independent of each other (Figure S1). Also, a feature importance plot (Figure 3) showed that the most important features were the tumor volume measured at baseline (Pre.T_morph.vol_) and the change in maximum distance between the tumor and involved lymph nodes measured at two time points (Δ.N_morph.spread2_), followed by the nodal spread measured before RT (Pre. N_morph.spread1_). The features extracted from lymph nodes, including Pre.N_bound.std_ and nodal volume differences between the pre-RT and mid-RT (Δ.N_morph.vol_), had important prognostic value, ranking 4^th^ and 5^th^, respectively. Moreover, the mid-RT nodal-tumor spread (Mid. N_morph.spread2_), Mid.N_ih.entropy_, and delta radiomics features of node number (Δ.N_morph.num_) and nodal spread (Δ.N_morph.spread1_) were important radiomic features, suggesting great value of mid-RT imaging features in predicting patient outcomes. The final imaging signature contained three features, including Pre. T_morph.vol_, Δ. N_morph.spread2_ and Pre. N_morph.spread1_ (see their detailed definition in Table 3), each of which were significantly associated with PFS (*P* < 0.05).

### Performance of the imaging signature in predicting PFS

**Figure 4:**
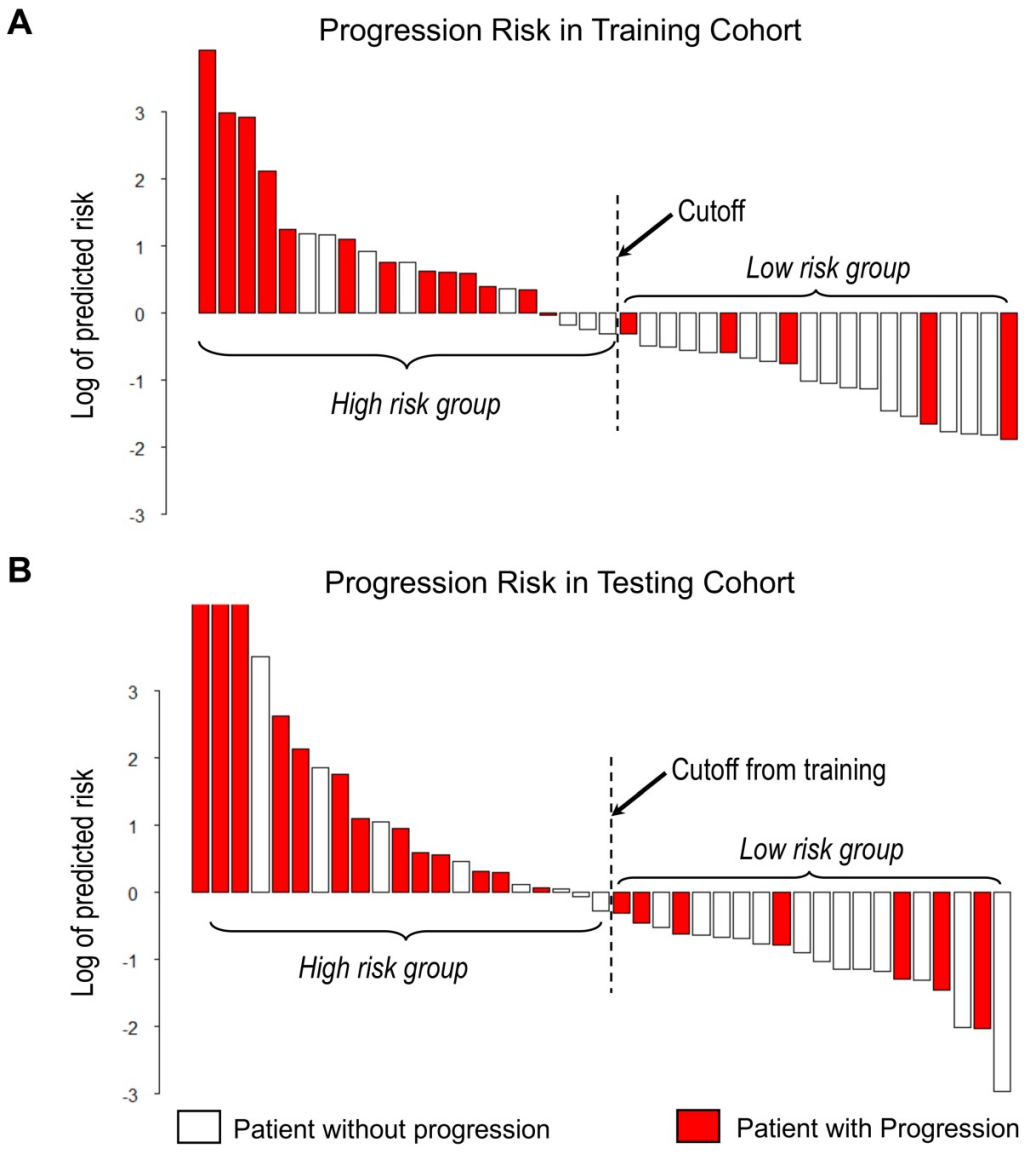
Waterfall plot of predicted risk of PFS according to the proposed imaging signature for A) Training cohort and B) Testing cohort.

**Figure 5:**
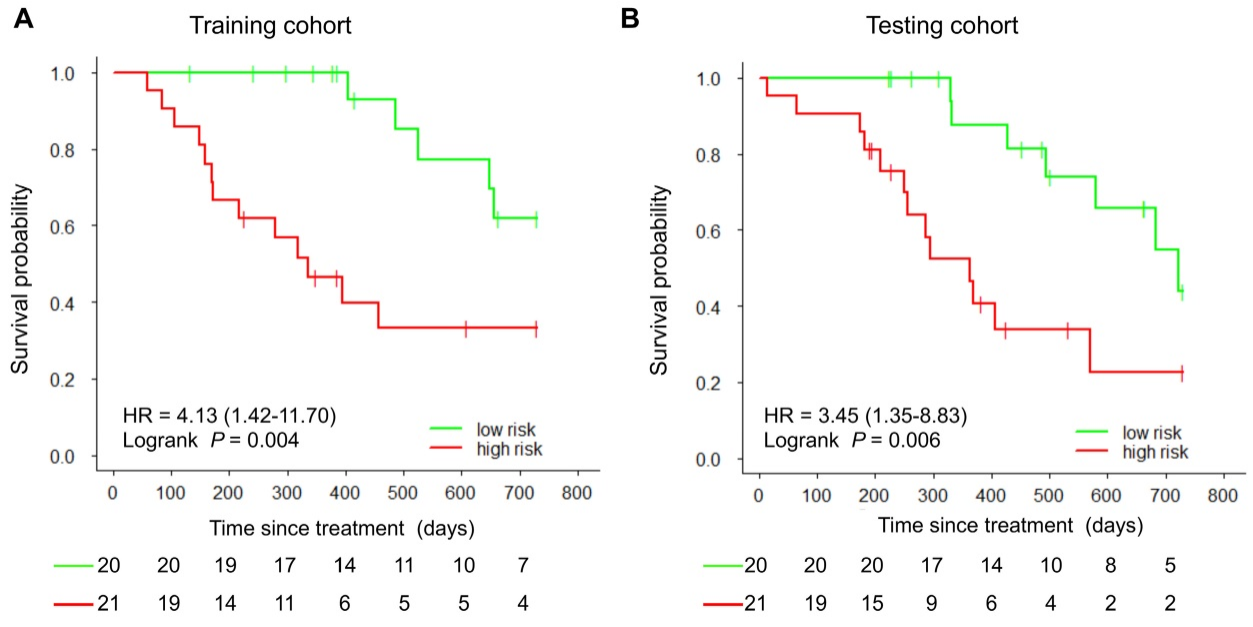
** Kaplan-Meier curves of PFS in the study patients.** At the cut-off value in the imaging model, patients were stratified into low-risk and high-risk groups regarding disease progression. A) Training cohort. B) Testing cohort.

With the median as the cut-off value (Figure 4A), the imaging signature stratified 41 patients in the training cohort into two groups at low and high risk for disease progression (*P* = 0.004 [log-rank test]; Figure 5A). The two patient groups had significantly different prognoses. Specifically, the 2-year PFS rates were 61.9% in the low-risk group and 33.2% in the high-risk group. Also, the median PFS duration was 19.3 months in the low-risk group and 10.5 months in the high-risk group.

Using the same cut-off value, we independently validated the imaging model in the testing cohort stratified into low- and high-risk groups (Figure 4B). The results showed that the patients in the low and high-risk groups had significantly different PFS (*P* = 0.006 [log-rank test]; HR, 3.45, [95% CI, 1.35-8.83]; Figure 5B). The 2-year PFS rate in the low-risk group was 43.8%, whereas that in the high-risk group was 22.6%. Also, the median PFS duration was 16.4 months in the low-risk group and 9.5 months in the high-risk group.

### The proposed imaging signature outperformed ablated models based on solely pre-RT or mid-RT PET/CT features

Similarly, we built two ablated imaging signatures from features extracted from pre-RT or mid-RT images, where the feature importance was shown in Figure S2. For the ablated model based on pre-RT features, nodal spread, tumor volume, and node boundary sharpness were the three most important ones. In contrast, for the ablated model from mid-RT features, node-tumor spread, tumor volume, and nodal spread were the three most important ones. The proposed imaging signature achieved higher prediction accuracy than two ablated models for predicting PFS (Figure S3). Further, the performance of individual selected features in the proposed imaging signature for predicting PFS was shown in Figure S3.

### The proposed imaging signature outperformed conventional clinical and imaging markers in predicting PFS

We compared the performance of the proposed imaging model and TNM stage (IIIA vs. IIIB) in classifying 2-year PFS, the resulting receiver operating characteristic curves for which are presented in Figure S4. In both the training and testing cohorts, the imaging model better predicted disease progression (AUC, 0.74 or 0.71) than did the TNM stage (AUC, 0.66 or 0.60). Furthermore, in a univariate analysis, the proposed imaging signature outperformed available clinical factors, including age, sex, histological type, and KPS (Table 4). During validating in the testing cohort, the imaging signature-based risk score remained significantly associated with PFS (HR, 1.40 [95% CI, 1.04-1.88]; *P* = 0.027).

Moreover, in the multivariate Cox regression, the imaging model was an independent predictor of PFS in the training cohort (HR, 1.14 [95% CI, 1.04-1.24]; *P* = 0.003) adjusting for available clinical factors (Table 4). In contrast, patient age, sex, TNM stage, and KPS were not significantly associated with PFS. We observed a similar trend in the testing cohort, as the imaging signature demonstrated a significant association of PFS (HR, 1.21 [95% CI, 1.10-1.33]; *P* = 0.048).

**Figure 6:**
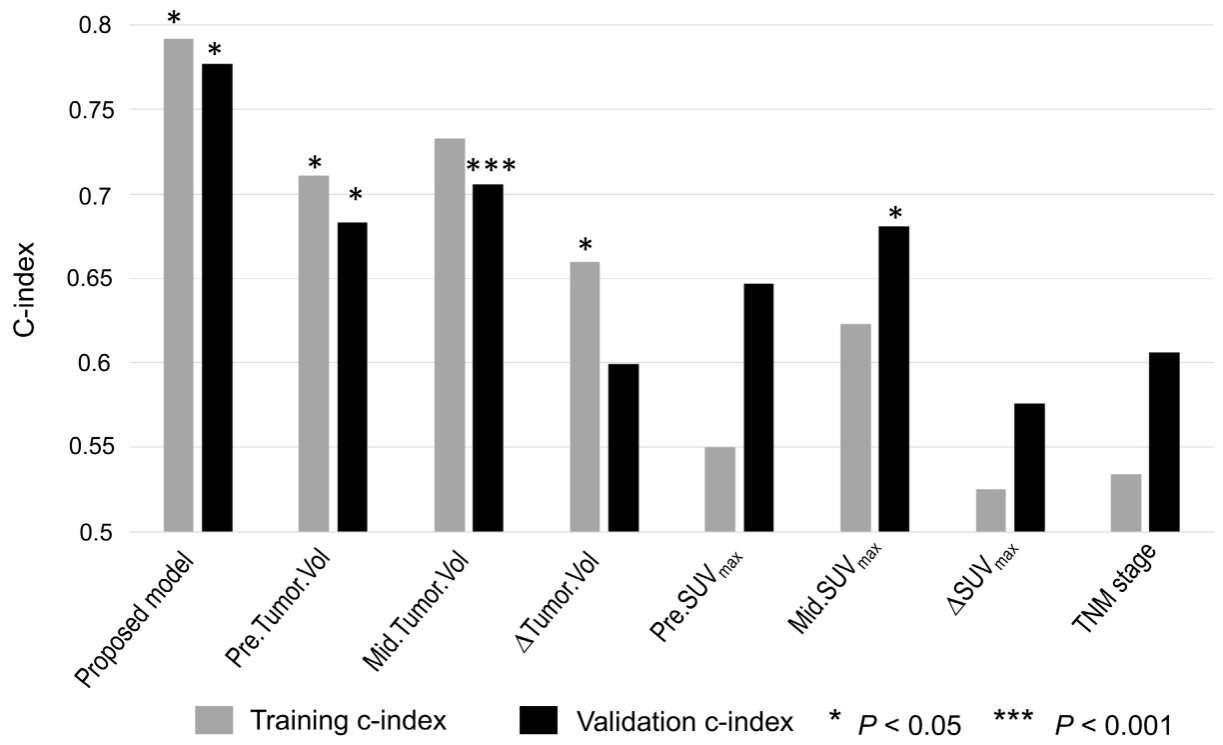
** Accuracy of predicting PFS as measured using the C-index for the imaging signature compared with six conventional imaging features and one clinical parameter.** The conventional imaging features were pre-RT tumor volume, mid-RT tumor volume, change in tumor volume, pre-RT SUV_max_, mid-RT SUV_max_, and change in SUV_max_. The clinical parameter was TNM stage.

As shown in Figure 6, the proposed imaging signature outperformed conventional PET/CT metrics, including tumor volume and SUV_max_, and the TNM stage in both the training and testing cohorts, with C-indices of 0.79 and 0.76, respectively. In particular, the proposed imaging signature achieved a markedly higher accuracy than did mid-RT tumor volume, which was the single best predictor of PFS among the selected conventional imaging features.

## Discussion

In this study, we constructed a prognostic PET/CT imaging signature in stage III NSCLC patients using a quantitative radiomics approach. In particular, we crafted new features to characterize tumor spread through analysis of the involved lymph nodes as well as to assess early treatment response by computing feature variations (i.e., delta radiomics) between baseline and mid-treatment scans. Based on these features, we developed a PET/CT radiomic signature that can stratify patients into distinct progression risk groups. We further demonstrated that the newly derived PET/CT model outperformed and complemented known clinical risk factors, including age, sex, TNM stage, and KPS. Another strength of our study is that we focused on stage III NSCLC patients who underwent similar RT treatments to mitigate potential confounding effects. With future validation, this imaging-based prognostic model may play a role in optimizing treatment intensity for locally advanced NSCLC patients with a high risk of disease progression.

To the best of our knowledge, this is the first study investigating the use of quantitative PET/CT features of both tumors and lymph nodes at pre-RT and mid-RT time points to predict the risk of disease progression in patients with stage III NSCLC. Previous PET radiomic studies of a similar population 12, 25-27 focused only on primary tumors and quantified few standard features, such as SUV_max_ and MTV. Lymph nodes are frequent sites of regional metastasis and represent critical information for cancer staging. Indeed, pilot studies proved that radiomic features of lymph nodes demonstrated superior prognostic value over those of the primary tumors in lung or head and neck cancer patients 23, 28. In particular, the number and dispersed distance of involved lymph nodes were informative of NSCLC patients' survival 29, 30. Our study corroborated these reported clinical values associated with lymph nodes, as nodal features make up four out of five top-ranked important features (Figure 3). Moreover, our PET/CT model has two nodal spread features that complement primary tumor volume, and combining them in the final imaging signature augmented PFS-predictive accuracy.

Also, we did not observe an association between the nodal volumetric burden and tumor progression, which was consistent with previous findings 29, 31.

The negative results of the RTOG 0617 multicenter trial described above highlighted the unmet need for effective prognostic markers to stratify patients with inoperable stage III NSCLC for individualized management. To address this, we examined the uniform cohort of 82 stage III NSCLC patients described herein, who were similar to the patients enrolled in RTOG 0617. Moreover, our patients received definitive chemoradiation using consistent, highly conformal techniques. Our proposed imaging signature achieved good performance for predicting tumor progression, outperforming conventional PET features and the TNM stage. Potentially, this signature can stratify stage III NSCLC patients into high and low-risk groups requiring different treatment strategies.

Our study contained the largest number of patients used to examine the clinical value of serial PET/CT scans at baseline and in the middle of RT of stage III NSCLC. Most previous radiomics studies focused on analyzing pre-treatment PET scans to characterize the metabolic heterogeneity of only primary tumors 22, 32-37. Several pilot studies have investigated serial PET images acquired both at baseline and during treatment. Gensheimer et al. showed that pre-RT MTV and mid-RT MTV and total lesion glycolysis (TLG) were positively associated with local recurrence of stage III NSCLC 13. Also, Yossi et al. showed that early assessment of TLG response via mid-RT PET was associated with survival 38. Dong et al. reported that early changes in PET textural features might be valuable for predicting treatment response and survival of locally advanced NSCLC 39. Building on but different from these studies, we proposed a set of forty-five quantitative imaging features that comprehensively describe both tumors and lymph nodes separately at pre-RT and mid-RT PET scans as well as the temporal changes between the two serial scans. Moreover, the extracted features are non-redundant, and all of them can be clearly interpreted. We found that mid-RT scans provided key information to complement features extracted from pre-RT images. In the proposed imaging signature, one out of three finally selected features was from mid-RT scans to describe the change in nodal spread as an early treatment response measure. One important advantage of our PET/CT model is that it relies on contour-based features and thus may be less dependent on the exact value of PET SUV intensity. In contrast, many features used in previous radiomic studies, such as histogram and texture, are sensitive to variations in PET SUV intensity, which can be caused by a variety of acquisition factors as well as reconstruction and value normalization algorithms.

Our study had some limitations. First, we studied a relatively small number of patients, which may have reduced the statistical power. However, to the best of our knowledge, our study had the largest number of patients in an investigation of quantitative PET/CT features of both primary tumors and lymph nodes at pre-RT and mid-RT time points for predicting the risk of disease progression in patients with stage III NSCLC. Second, it was a retrospective study with potential confounding factors. We followed a strict training and independent testing scheme by evenly partitioning the overall cohort in training and testing cohorts, which further reduced the sample size in the training cohort. Also, all enrolled patients were treated at a single research institution. However, although the PET scans were acquired using two scanners, they followed similar imaging protocols. Third, we included patients with two major histological subtypes of NSCLC: adenocarcinoma and squamous cell carcinoma. Due to the small sample size, we did not carry subgroup analysis and separate patients according to their histologic subtypes. Fourth, Steinfort and colleagues found that systematic node staging by EBUS-TBNA can detect PET-occult LN metastases in 5-10% of NSCLC patients 40. Therefore, accurate lymph node staging by EBUS can potentially improve the performance of the imaging signature.

In the future, prospective multicenter validation of our proposed imaging signature will be needed to further confirm its prognostic value and potential role in guiding personalized RT for stage III NSCLC. In addition, the underlying biological mechanisms explaining the prognostic value of our imaging signature should be investigated. Integrating corresponding biological information on multiple spatial scales, such as molecular, cellular and tissue levels, we can explore the drivers behind high-risk patients as predicted by the PET imaging model using radiogenomic frameworks 41. As more information on tumor is collected including molecular markers of NSCLC 42, 43, combining biospecimen-derived information with this imaging signature may achieve the highest level of accuracy in NSCLC patient risk stratification.

In conclusion, we constructed a quantitative imaging signature combining primary tumor and nodal imaging features from baseline and mid-RT PET/CT scans to predict disease progression in patients with stage III NSCLC. This imaging signature is a method of non-invasive evaluation of the dynamic phenotypes of NSCLC that has great potential for guiding individualized treatment of this disease.

## Supplementary Material

Supplementary figures.

## Figures and Tables

**Table 1:** Forty-five radiomic features extracted from patients' PET/CT scans.

Tumor at pre- and mid-RT (n=22)	Lymph Node at pre- and mid-RT (n=18)	Δ Features, mid - pre (n=5)
Morphology	Morphology	Tumor
Volume (T_morph.vol_)	Volume (N_morph.vol_)	Δ Volume (Δ. T_morph.vol_)
Sphericity (T_morph.sphericity_)	Number (N_morph.num_)	Lymph Node
Boundary Sharpness	Nodal Spread (N_morph.spread1_)	Δ Volume (Δ.N_morph.vol_)
Mean (T_bound.mean_)	Node-Tumor Spread (N_morph.spread2_)	Δ Number (Δ. N_morph.num_)
Standard Deviation (T_bound.std_)	Boundary Sharpness	Δ Nodal Spread (Δ. N_morph.spread1_)
Intensity	Mean (N_bound.mean_)	Δ Node-Tumor Spread (Δ. N_morph.spread2_)
Mean (T_ih.mean_)	Standard Deviation (N_bound.std_)	
Standard Deviation (T_ih.std_)	Intensity	
Entropy (T_ih.entropy_)	Mean (N_ih.mean_)	
GLCM Texture	Standard Deviation (N_ih.std_)	
Contrast (T_cm.contrast_)	Entropy (N_ih.entropy_)	
Homogeneity (T_cm.homogeneity_)		
Correlation (T_cm.corr_)		
Energy (T_cm.energy_)		

**Table 2:** Demographic and clinical characteristics of the study patients

Parameter	Training (n=41)	Testing (n=41)	*P* value
**Age (years)**	**68.2 (41.5-89.2)**	**67.9 (47.2-89.7)**	**0.253**
**Sex**			**0.736**
Male	25 (61.0%)	23 (56.1%)	
Female	16 (39.0%)	18 (43.9%)	
**T Stage**			**0.121**
T1	12 (29.2%)	9 (22.0%)	
T2	9 (22.0%)	15 (36.6%)	
T3	5 (12.2%)	11 (26.8%)	
T4	15 (36.6%)	6 (14.6%)	
**N Stage**			**0.493**
N0	2 (4.9%)	1 (2.4%)	
N1	2 (4.9%)	2 (4.9%)	
N2	24 (58.5%)	21 (51.2%)	
N3	13 (31.7%)	17 (41.5%)	
**TNM Stage**			**0.732**
IIIA	20 (48.8%)	18 (43.9%)	
IIIB	21 (51.2%)	23 (56.1%)	
**Histology**			**0.213**
Adenocarcinoma	18 (43.9%)	18 (43.9%)	
SCC	16 (39.0%)	10 (24.4%)	
NSCLC-NOS	7 (17.1%)	13 (31.7%)	
KPS Score	80 (60-100)	80 (50-100)	**0.237**
**PFS**			**0.942**
Event	20 (48.8%)	21 (51.2%)	
No event	21 (51.2%)	20 (48.8%)	
**Follow-up (year)**			**0.659**
Median, std	2.0 (0.8)	1.9 (0.6)	

**Table 3:** Details of the three imaging features in the final cox model for predicting PFS

Selected PET features	HR	95% CI	*P* value
Baseline tumor volume (**Pre. Tmorph.vol**)	5.23	2.04 - 13.41	<0.001
Change in maximum distance between the primary tumor and involved nodes measured at two time points (**Δ. Nmorph.spread2**)	2.19	1.25 - 3.86	0.007
Baseline maximum distance between involved nodes (**Pre. Nmorph.spread1**)	1.99	1.15 - 4.44	0.014

**Table 4:** Results of univariate and multivariate analyses of the proposed imaging signature and clinical factors in predicting PFS

Predictors	Training cohort						Testing cohort					
	Univariate			Multivariate			Univariate			Multivariate		
	HR	95% CI	*P* value	HR	95% CI	*P*-value	HR	95% CI	*P*-value	HR	95% CI	*P* value
**Imaging model**	1.14	1.03 - 1.27	0.013 *	1.14	1.04 - 1.24	0.003 *	1.40	1.04 - 1.88	0.027 *	1.21	1.10 - 1.33	0.048 *
**Age**	1.03	0.98 - 1.08	0.248	1.03	0.98 - 1.08	0.275	0.97	0.93 - 1.02	0.240	0.97	0.92 - 1.03	0.281
**Sex**	0.96	0.40 - 2.31	0.921	1.75	0.58 - 5.26	0.323	0.83	0.35 - 1.98	0.674	0.78	0.27 - 2.24	0.643
**Stage**	1.49	0.62 - 3.57	0.376	1.23	0.42 - 3.57	0.699	1.39	0.62 - 3.33	0.424	1.32	0.21 - 1.47	0.236
**Histology**	1.37	0.54 - 3.47	0.511	2.06	0.70 - 6.03	0.187	1.17	0.48 - 2.84	0.725	1.85	0.68 - 5.04	0.230
**KPS**	0.96	0.91 - 1.00	0.068	0.97	0.92 - 1.02	0.266	0.96	0.92 - 1.01	0.132	0.97	0.92 - 1.02	0.243

Note: 1. male as 1, female as 0; 2. IIIA as 0, IIIB as 1; 3. KPS as continuous value; 4. Adenocarcinoma as 1, others as 0.

## Abbreviations

CI: confidence interval
CT: computed tomography
18F-FDG: 18F-fluorodeoxyglucose
HR: hazard ratio
KPS: Karnofsky performance score
MTV: metabolic tumor volume
NSCLC: non-small cell lung cancer
PET: positron emission tomography
PFS: progression-free survival
RT: radiotherapy
SUV_max_: maximum standardized uptake value
