# Supplementary material for: Early response evaluation using primary tumor and nodal imaging features to predict progression-free survival of locally advanced non-small cell lung cancer
Source: Theranostics. 2020 Sep 23;10(25):11707–18. doi: 10.7150/thno.50565 (PMC7546006; doi:10.7150/thno.50565)
Supplement: Supplementary file 1 — Supplementary figures. [file thnov10p11707s1.pdf]

Supplementary figures

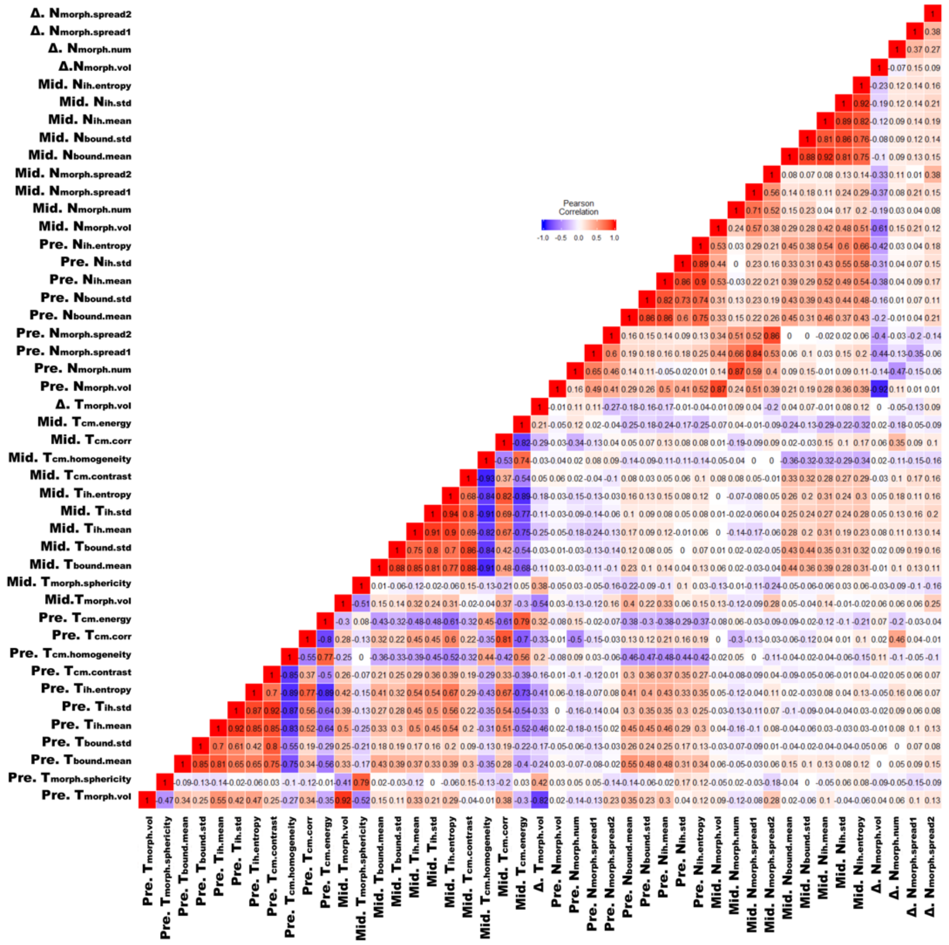

Figure S1. Pair-wise Pearson's correlation of 45 quantitative radiomics features.

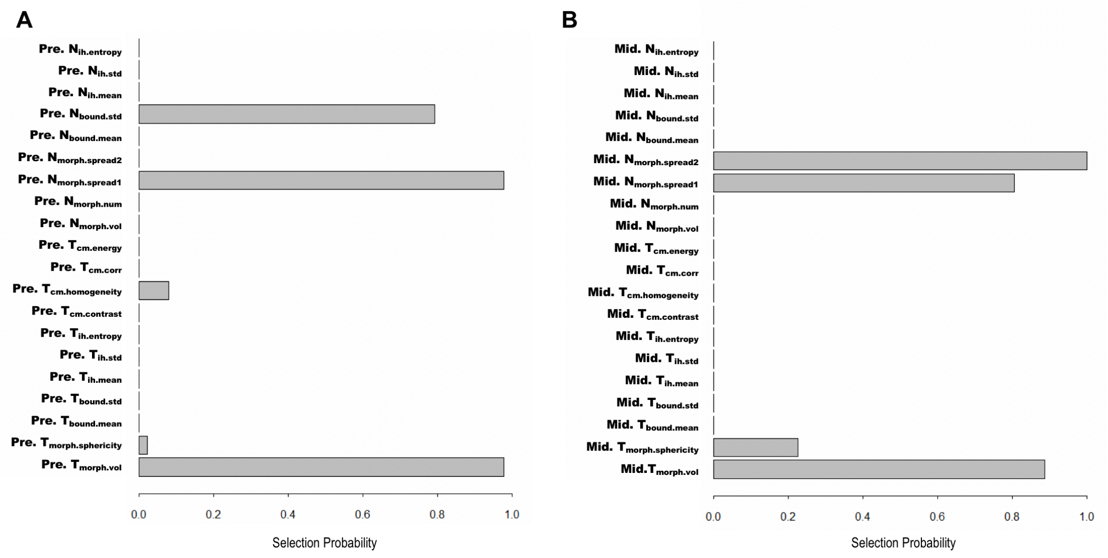

Figure S2. Two ablated imaging signatures from features extracted from A) pre-RT or B) mid-RT images. The feature importance was shown.

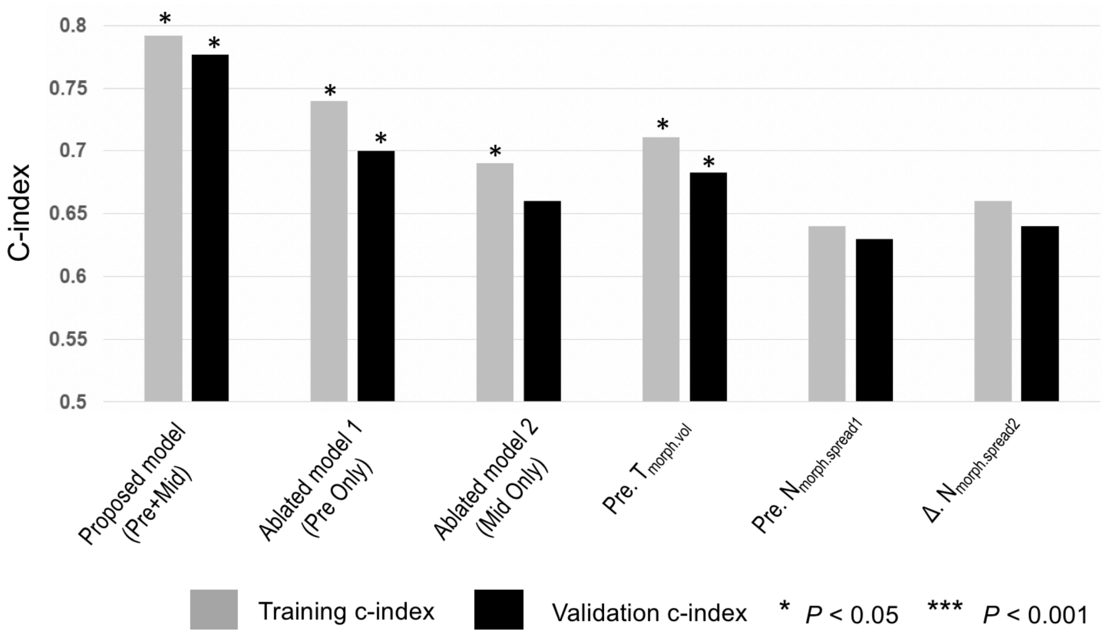

Figure S3. Accuracy of predicting PFS as measured using the C-index for the imaging signature compared with two ablated models. And the performance of individual selected features in the proposed imaging signature for predicting PFS were shown.

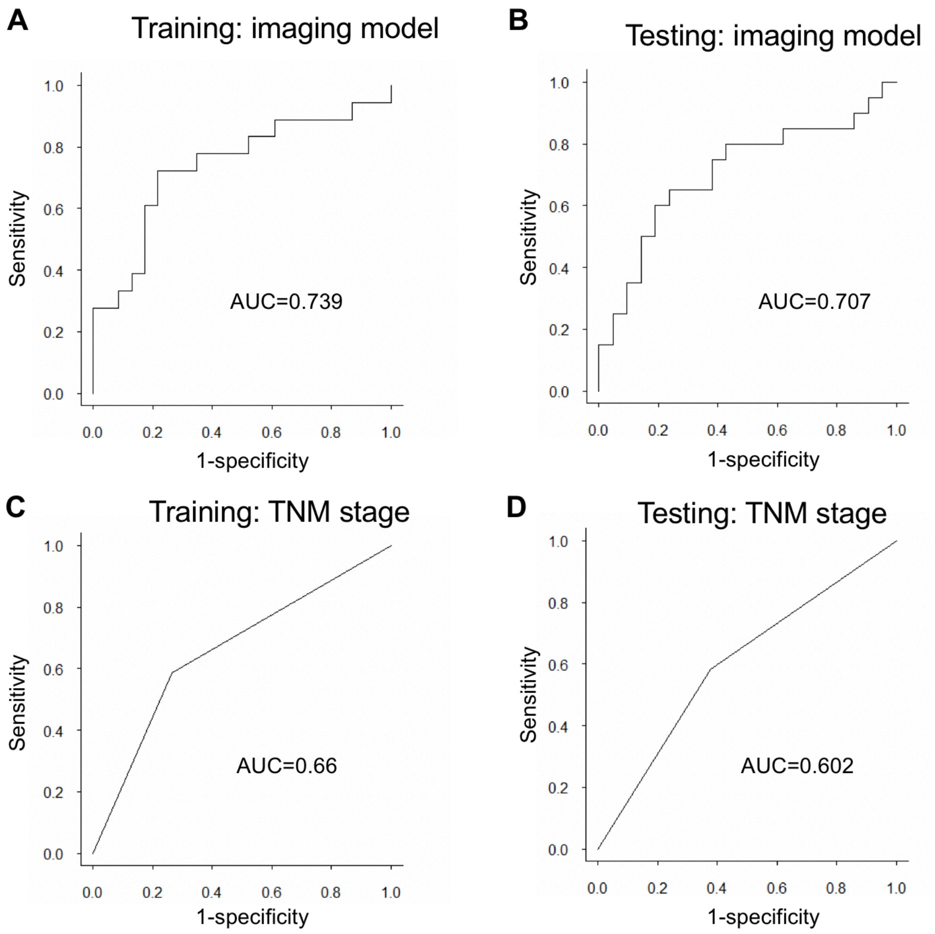

Figure S4. Evaluation of the predictive performance of the imaging model and TNM stage.
